# Supplementary material for: XPF–ERCC1 Blocker Improves the Therapeutic Efficacy of 5-FU- and Oxaliplatin-Based Chemoradiotherapy in Colorectal Cancer
Source: Cells. 2023 May 25;12(11):1475. doi: 10.3390/cells12111475 (PMC10252687; doi:10.3390/cells12111475)
Supplement: Supplementary file 1 [file cells-12-01475-s001.zip › cells-2342115-supplementary.pdf]

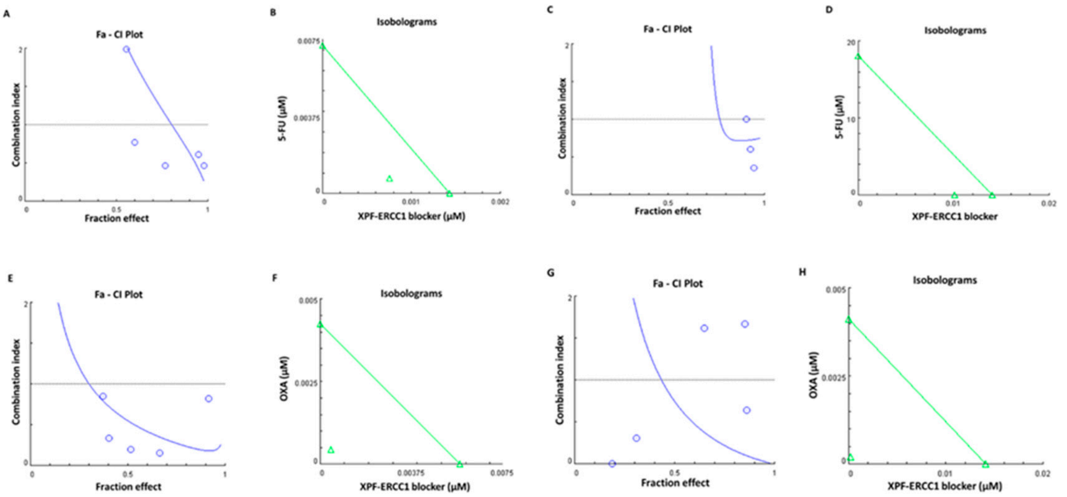

Figure S1. Dose effect relationship of XPF-ERCC1 blocker, OXA, and 5-FU combination in rectal cancer cell lines Combined 5-FU and XPF-ERCC1 blocker in HCT116 (A) and SW620 (C) cells. Combined OXA and XPF-ERCC1 blocker in HCT116 (E) and SW620 (G) cells. FaCI plots were obtained from median effect analysis (Calcusyn). Solid lines show computer simulated FaCI plots. Circles represent experimental data points. CI<1, CI=1, and CI>1 indicate synergism, additive effects, and antagonism, respectively. Isobolograms indicate the nature of the combined 5-FU and XPF-ERCC1 blocker, or the combined OXA and XPF-ERCC1 blocker at constant ratios in HCT116 (B, D) or SW620 (F, H). Respective drug combinations at the ED50 effect levels; data points below the line = synergistic, on the line = additive, and above the line = antagonistic effects. The degree of synergism in this drug combination is reflected by the distance of the data point from its respective line (same color). Fa, fraction affected; CI, combination index; OXA, oxaliplatin; ED50, median effective dose needed to inhibit 50% of cells. All experiment was independently repeated four times.

Table S1. Cytotoxic effects of 5-FU, OXA and XPF-ERCC1 blocker in colorectal cancer lines.

**Half-maximal inhibitory concentration, μM**		
**Compounds**	**HCT116**	SW620
5-FU	29.10	57.57
Oxaliplatin (OXA)	2.62	69.39
XPF-ERCC1 bolcker	41.86	60.85
5-FU / ERCC1-XPF blocker	12.46	21.41
OXA/ ERCC1-XPF blocker	1.46	17.76
